# A Phase I study of the angiogenesis inhibitor SU5416 (semaxanib) in solid tumours, incorporating dynamic contrast MR pharmacodynamic end points

**DOI:** 10.1038/sj.bjc.6602797

**Published:** 2005-10-11

**Authors:** A O'Donnell, A Padhani, C Hayes, A J Kakkar, M Leach, J M Trigo, M Scurr, F Raynaud, S Phillips, W Aherne, A Hardcastle, P Workman, A Hannah, I Judson

**Affiliations:** 1 Institute of Cancer Research, Sutton SM2 5NG, UK; 2 Royal Marsden Hospital, Sutton SM2 5PT, UK; 3 Paul Strickland Scanner Centre, Mount Vernon Hospital, Rickmansworth Road, Northwood, Middlesex, UK; 4 Thrombosis Research Institute, Imperial College, London, UK; 5 Sugen Inc., South San Francisco, CA, USA

**Keywords:** antiangiogenic therapy, phase I clinical trial, pharmacokinetics, pharmacodynamics

## Abstract

SU5416 (Z-3-[(2,4-dimethylpyrrol-5-yl)methylidenyl]-2-indolinone; semaxanib) is a small molecule inhibitor of the vascular endothelial growth factor receptor (VEGFR)2. A Phase I dose escalation study was performed. Dynamic contrast-enhanced magnetic resonance imaging (DCE-MRI) was used as a pharmacodynamic assessment tool. In all, 27 patients were recruited. SU5416 was administered twice weekly by fixed rate intravenous infusion. Patients were treated in sequential cohorts of three patients at 48, 65, 85 110 and 145 mg m^−2^. A further dose level of 190 mg m^−2^ after a 2-week lead in period at a lower dose was completed; thereafter, the cohort at 145 mg m^−2^ was expanded. SU5416 showed linear pharmacokinetics to 145 mg m^−2^ with a large volume of distribution and rapid clearance. A significant degree of interpatient variability was seen. SU5416 was well tolerated, by definition a maximum-tolerated dose was not defined. No reproducible changes were seen in DCE-MRI end points. Serial assessments of VEGF in a cohort of patients treated at 145 mg m^−2^ did not show a statistically significant treatment-related change. Parallel assessments of the impact of SU5416 on coagulation profiles in six patients showed a transient effect within the fibrinolytic pathway. Clinical experience showed that patients who had breaks of therapy longer than a week could not have treatment reinitiated at a dose of 190 mg m^−2^ without unacceptable toxicity. The 145 mg m^−2^ dose level is thus the recommended dose for future study.

SU5416 (Z-3-[(2,4-dimethylpyrrol-5-yl)methylidenyl]-2-indolinone; semaxanib) has been developed as a potent inhibitor of the receptor tyrosine kinase for vascular endothelial growth factor receptor – Flk-1/KDR (VEGFR2) (Figure 1). This receptor pathway plays a crucial role in the process of angiogenesis. Targeting angiogenesis is highly attractive as a strategy to control malignancy. Such a therapy should be less susceptible to the development of resistance, since the target is normal endothelial cells, and as the majority of normal tissue is not actively developing vasculature, it should not be accompanied by the toxicity of conventional cytotoxics.

SU5416 is also a potent competitive inhibitor of KIT (the receptor for stem cell factor) and less potently the PDGF receptor (indirectly involved in angiogenesis), but is not directly cytotoxic by itself (Antonian *et al*, 2000; Smolich *et al*, 2001; Yee *et al*, 2002). Recent work has also suggested that SU5416 may mediate some of its effects through inhibition of a second VEGF receptor – Flt-1 (VEGFR1) (Itokawa *et al*, 2002). Preclinical xenograft models have confirmed inhibition of tumour growth and a reduction in the number of metastases following treatment with SU5416 (Fong *et al*, 1999), as well as a decrease in vascular density in treated tumours as measured by intravital video-microscopy (Vajkoczy *et al*, 1999; Vaskoczy *et al*, 2000).

Theoretically, an antiangiogenic treatment may prove initially cytostatic rather than cytoreductive. As such, chronic therapy may be required, issues of toxicity and compliance become increasingly important and it would be clinically desirable if one could identify responding patients at the earliest juncture. Thus ideally in preclinical investigations, but certainly in the Phase I clinical setting, it is critical to validate noninvasive pharmacodynamic tools that indicate a dose-dependent biological effect.

In this Phase I trial, the primary objective was to assess the safety and tolerability of SU5416 when administered twice weekly via intravenous infusion. Secondary objectives were to quantify effects on tumour vascular permeability using low-molecular weight dynamic contrast-enhanced magnetic resonance imaging (DCE-MRI), to assess the pharmacokinetics (PK) of SU5416 and to correlate PK with DCE-MRI. This use of imaging to assess vascular permeability was designed to investigate SU5416 pharmacodynamics in humans for the first time in a structured dose escalation manner, in an effort to determine an effective dose for further development. It is thus unique among parallel Phase I studies with this compound hitherto reported (Rosen *et al*, 1999; Stopeck *et al*, 2002). In addition, effects on coagulation and VEGF levels were explored and any evidence of antitumour activity was reported.

## PATIENTS AND METHODS

### Clinical protocol

The study protocol was approved by the Research Ethics Committee of the Royal Marsden NHS Trust. Written informed consent was obtained from all participants. All patients had treatment refractory solid tumours and were required to have at least one tumour deposit suitable for DCE-MRI evaluation. A suitable lesion had to be ⩾2 cm in size and in an anatomical position not subject to significant respiratory excursion. Eligible patients had not received treatment with any other anticancer therapy for 4 weeks prior to treatment with SU5416. Patients were required to have adequate baseline haematological reserves (absolute neutrophil count ⩾1.5 × 10^9^ l^−1^, platelet count ⩾100 × 10^9^ l^−1^, haemoglobin ⩾9.0 g dl^−1^). In addition, all patients were required to have adequate systemic organ function (i.e. serum transaminases <2.5 times the upper limit of normal; total bilirubin <34.2 *μ*mol l^−1^; and serum creatinine <160 *μ*mol l^−1^ or creatinine clearance ⩾50 ml min^−1^). Exclusion criteria included patients with a known history of allergy to radiologic contrast agents or to Cremophor®; a Karnovsky performance status of <60%; a history of myocardial infarction, severe or unstable angina within 6 months of the study; diabetes mellitus with clinical evidence of severe peripheral vascular disease or ulceration; a medical condition or device implant that would interfere with or prevent MRI scans; or a serious concomitant medical condition that could interfere with the participation in the study or interpretation of study results.

The starting dose of SU5416 for the study was 48 mg m^−2^. This dose was chosen in the light of available clinical experience in a concomitant Phase I trial underway in Los Angeles. Sequential cohorts of three patients were recruited. Dose escalation steps of 33% were planned. The National Cancer Institute Common Toxicity Criteria V1 was used to grade toxicity. Dose-limiting toxicity (DLT) was defined as the occurrence of: Grade III or greater toxicity excluding nausea/vomiting and haematologic toxicity; or Grade IV haematologic toxicity; or Grade IV nausea and vomiting refractory to antiemetic therapy. When DLT was observed in one patient in a cohort, the dose level was expanded to six patients. The maximum-tolerated dose in this study was defined as the dose level below which DLT occurred in ⩾2 of the six patients treated in the cohort. Intrapatient dose escalation was permitted.

SU5416 was administered twice weekly via intravenous infusion at a fixed rate of 200 ml h^−1^. All patients received intravenous chlorpheniramine (10 mg), dexamethasone (10 mg) and ranitidine (50 mg) as premedication 1 h before treatment, to minimise hypersensitivity reactions attributable to the excipient Cremophor®.

Patients were monitored for at least 1 h after treatment. Samples for haematology, coagulation studies, biochemistry and urine analysis were taken on day 15 and four weekly thereafter. Full tumour re-evaluation was performed every 6 weeks using WHO response criteria. Patients were allowed to continue to receive therapy with SU5416 indefinitely in the absence of unacceptable toxicity or evidence of tumour progression.

### Study drug – SU5416

SU5416 (SUGEN Inc., South San Francisco, CA, USA) was supplied as a yellow-orange liquid formulation in 30 ml vials each containing 112.5 mg of drug in 25 ml of vehicle for a final concentration of 4.5 mg ml^−1^. Vehicle constituents in the formulation included: polyethylene glycol 400; polyoxyl 35 castor oil (Cremophor®); and benzyl alcohol and dehydrated alcohol. The vials were stored at room temperature (22–28°C) and were protected from light. Prior to administration, SU5416 was diluted 1 : 3 with 0.45% sodium chloride and injected into non-PVC intravenous infusion bags. Treatment infusions were administered through non-PVC, non-DEHP intravenous tubing and a 0.2 *μ*m in-line filter (not made from cellulose acetate).

### PK evaluation

Plasma samples for the determination of SU5416 and its metabolites were drawn on day 4 and day 25. Samples were taken prior to treatment infusion, 15 min into the infusion, at completion of the infusion and then at 5, 10, 20, 30, 45, 60, 120 and 240 min following the infusion. Blood samples were drawn from the contralateral arm or distant from the venous access device used for the administration of SU5416. At the designated times, 4.0 ml of blood were collected into a lithium heparin tube, gently inverted and placed on ice to maintain the sample at 2–8°C. All samples were immediately centrifuged at 6000 r.p.m. at 4°C for 10 min, the supernatant divided between two 1.5 ml Nalgene cryovials and snap frozen in liquid nitrogen. Separated plasma samples were subsequently stored at −70°C until analysis.

Pharmacokinetic analysis was performed using a fully validated HPLC UV method (Stopeck *et al*, 2002). Plasma samples, standards (25–2000 ng ml^−1^) and quality controls spiked with internal standards were extracted with acetonitrile. Following centrifugation, drying and reconstitution in 200 *μ*l mobile phase, 20 *μ*l were analysed by HPLC UV. Chromatography was carried out using a C_18_ analytical column, with a gradient mobile phase containing acetonitrile and 10 mM ammonium formate at pH 2.6. The flow rate was 0.8 ml min^−1^ and the run-time 21 min. Detection was via UV absorption at a wavelength of 440 nm. The accuracy of the assay, reflected in relative error of measured concentration to theoretical (prepared) concentrations, was between 0.9 and 5.6% for all quality control samples.

Pharmacokinetic parameters were evaluated using WinNonLin Software®. A comprehensive PK profile of SU5416 and its metabolites SU6595 and SU9838 (*C*_max_, *T*_max_, *T*_1/2*α*_, *T*_1/2_ and *K*_abs_) was determined for each patient.

### Pharmacodynamic evaluations – VEGF

Assessment of VEGF levels was incorporated by amendment from the 145 mg m^−2^ dose level.

Samples for the measurement of VEGF levels were drawn at baseline, on day 1 at 2, 4 and 24 h, after the treatment infusion. At each time point, blood was drawn into a plain tube (serum), a sodium citrate tube (platelet-rich plasma) and a CTAD (platelet-stabilised plasma) tube. CTAD tubes (Becton-Dickinson Vacutainer Systems, Europe) contain four anticoagulants: sodium citrate, theophylline, adenosine and dipyridamole, and are designed to minimise any platelet activation that may occur as a result of blood collection.

Vascular endothelial growth factor was measured by enzyme-linked immunosorbent assay (ELISA) using the commercially available Quantikine Kit (R&D Systems, Europe). This analysis was conducted at The Institute of Cancer Research, Sutton, UK.

### Pharmacodynamic evaluation – DCE-MRI

All patients were asked to have a baseline DCE-MRI examination within the 7 days prior to receiving the study compound under study conditions as described above. Follow-up scans were performed on day 1 and then after 4 weeks of therapy. After the first three patients' examination, the protocol was amended to day 1, 2 weeks, 6 weeks and 6 weekly thereafter if the patient remained on study. All examinations were performed on a 1.5 T MRI system (Vision, Siemens Medical Systems, Erlangen, Germany) approximately 4 h after the commencement of the treatment infusion.

The image collection protocol comprised routine orthogonal T1- and T2-weighted images taken through the previously identified target lesion, to allow localisation and bi-dimensional tumour measurement. The dynamic imaging used a five-slice saturation recovery turbo fast low-angle shot sequence (SRTF) taken through the centre of the target lesion. Proton density-weighted images were acquired, followed by a series of T1-weighted images employing the same gain and image scaling factors. The sequential T1-weighted images were acquired every 9 s for 6.3 min (42 time points).

Gadopentetate dimeglumine (Magnevist Schering Health Care Limited, Burgess Hill, Sussex, UK) was injected intravenously using a mechanical power injector, through a peripherally placed cannula. This contrast was administered as a bolus after the third baseline data point was acquired at a dose of 0.1 mmol kg^−1^ body weight. The use of the mechanical power injector allowed the contrast to be delivered within 10 s. The contrast injection was followed by a 20 ml flush of normal saline.

Using the resultant scanned images, two trained observers (AP, CH) blinded to the clinical outcome of the patients evaluated the patterns of enhancement within a designated region of interest. Contrast medium kinetic parameters were then derived for each pixel using MR Imaging Workbench software®. Quantitative and semiquantitative statistical analysis was then performed on these parameters using StatsDirect software (Research Solutions, Cambridge, UK).

### Expanded coagulation series

During the dose escalation stage of the protocol, a standard series of coagulation parameters (APPT, INR and fibrinogen) was explored at baseline prior to therapy and every 2 weeks thereafter. In the dose expansion phase of the protocol, in response to the clinical occurrence of two separate venous thrombotic episodes in study patients, an expanded coagulation series was performed. Samples were taken on cycle 1, day 1 prior to, then at 4 and 24 h following the infusion of SU5416 and on cycle 1, day 4 and day 25 prior to, then 4 h after the SU5416 injection.

At each time point, blood was collected in citrated and EDTA tubes (Becton-Dickinson Vacutainer Systems, Europe). The samples from the citrated tubes were immediately centrifuged (at 4°C for 10 min at 3000 r.p.m.) supernatants pooled into inert plastic, centrifuged again (at 4°C, 10 min at 3000 r.p.m.) and the resultant sample stored in inert plastic tubes at −70°C until analysis. The sample taken into EDTA was centrifuged (at 4°C, for 10 min at 3000 r.p.m.), the supernatant decanted into inert plastic tubes and again stored at −70°C until analysis.

## RESULTS

### Patient characteristics and dose escalation

In all, 27 patients received treatment with SU5416 on this study. Descriptive details of the patients are shown in Table 1.

Sequential cohorts of three patients were treated at 48, 65, 85, 110, and then 145 mg m^−2^. In this dose escalation phase, a total of 24.5 cycles of SU5416 were administered (median per patient, 1.5; range 0.5–4). During the conduct of this trial, investigators in a parallel Phase I trial of SU5416 on the same schedule at The University of California, Los Angeles, USA reported DLT (projectile vomiting and severe headache) at 190 mg m^−2^ (Rosen *et al*, 1999). Pharmacokinetic studies in our trial (see below) had identified induction in the metabolism of SU5416 occurring within the first weeks of therapy. Therefore, it was decided to conclude the current study with a final dose expansion cohort of patients receiving 145 mg m^−2^ twice weekly for four doses, escalating the dose to 190 mg m^−2^ if well tolerated for subsequent therapy. In all, 12 patients were recruited to this expansion phase of the trial receiving in total 29.5 cycles of therapy (median 2.5; range 0.5–5).

### Pharmacokinetics

**Figure 2:**
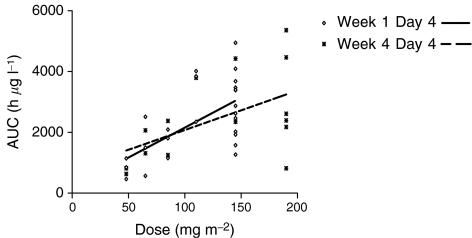
Pharmacokinetics: AUC *vs* dose demonstrating linearity and the induction of metabolism between week 1 and week 4.

The PK results for the 25 patients evaluable are presented in Table 2. SU5416 was well distributed with a large volume of distribution (*V*_d_ 39–215L). Plasma clearance was rapid (CL 46–215 l h^−1^), but with large interpatient variability for which there is no obvious explanation. SU5416 displayed linear kinetics over the dose range 48–145 mg m^−2^ (Figure 2) at initial assessment and also on day 25. However, the mean AUC at 190 mg m^−2^ in those patients escalated from 145 mg m^−2^ to the higher dose after 2 weeks did not differ significantly from that observed at 145 mg m^−2^ (3611±183 *vs* 3723±930 *μ*g l^−1^ h^−1^). A similar plateau is observed in mean peak concentration. (Table 2) In five of the nine patients who were dosed at the same level on both day 4 and day 25, there was an average increase in drug clearance of 22%.

SU6595 (the carboxyl derivative) was the predominant metabolite representing on average 78% of the parent, while SU9838 (the hydroxyl derivative) comprised only 5.2% of the total. Both metabolites behaved as the parent drug with a large volume of distribution and rapid clearance. Neither metabolite shows inhibitory activity for any of the receptor tyrosine kinases inhibited by the parent compound (Mendel *et al*, 2000). The increase in clearance observed for the parent compound between day 4 and day 25 was not associated with an increase in the plasma AUC of the main metabolite, SU6595 (data not shown). There was no correlation between toxicity and any of the PK parameters evaluated in this study.

### Toxicity

With the exception of hypersensitivity, the intravenous infusion of SU5416 was well tolerated. Despite premedication, Grade II hypersensitivity reactions affected 11 patients and occurred independent of the dose of SU5416. All patients were able to continue to receive therapy with increased corticosteroid cover. Tachyphylaxis did occur and the steroid dose was able to be gradually reduced in each patient affected.

Moderate thrombophlebitis was common when peripheral access was used. For those who continued with treatment for multiple cycles, central line access was recommended. Other acute toxicity included headache, fatigue, nausea and occasional vomiting. Such symptoms were mild, dose related and most commonly observed in the dose expansion cohort. Of interest, three patients in this cohort also reported pain or burning discomfort in tumour-related sites shortly after treatment infusions. Such symptoms were short lived and did not require intervention. Vital signs were monitored prior to and after treatment for the duration of the schedule. No predictable or persistent changes were observed. Two patients who received therapy for 6 weeks or more developed dysphonia. This did prove to be reversible over several months.

One patient (dose level 145 → 190 mg m^−2^, on study 20 weeks) developed recurrent Grade II anaemia that was felt to be related to SU5416. A further patient (dose level 145 → 190 mg m^−2^) developed a mild (Grade I) elevation in hepatic transaminases, which may have been due to study therapy, but this patient, with ovarian cancer, was known to have a disease plaque adjacent to the porta hepatis. These were, however, the only haematological and biochemical drug-related adverse events.

Venous thrombotic events occurred in two patients while they were receiving treatment with SU5416 (thrombosis of the inferior vena cava and a pulmonary embolism). Both patients had serious concomitant risk factors for such medical problems, and these events occurred at differing dose levels, but it is not possible to exclude an association with SU5416. The criteria for maximum-tolerated dose were not met. Of the remaining 26 patients who received SU5416 on this study, five were receiving low-dose warfarin for prophylaxis of central venous access lines and one patient was receiving therapeutic warfarin for hereditary coagulopathy.

### Coagulation

No change in INR, APTT or fibrinogen was observed. In all six patients evaluated using the expanded panel, a consistent acute increase in plasminogen activator inhibitor (PAI-1) levels was observed with recovery at 24 h (median change 37.3 IU; range 14.23–61–27). A parallel reduction in plasmin–anti-plasmin complexes (median change 12.7 *μ*g l^−1^; range 0–36) was also seen. There were no consistent changes in the coagulability parameters: anti-thrombin III, protein C (act), protein S (free), activated protein C resistance, dRVVT, endogenous thrombin (generation) potential (extrinsic and intrinsic), prothrombin fragments 1 and 2 and thrombin–anti-thrombin complexes. No change was seen in the endothelial cell haemostatic properties: thrombomodulin or Von Willebrand factor (activated and agglutinated).

### Tumour response

Two patients were not evaluable for response because major intercurrent illness, unrelated to therapy with SU5416 occurring after <2 weeks of therapy, precluded disease assessment (pneumonia, bowel obstruction). In all, 12 patients were considered to have stable disease at 6 weeks and went on to receive further treatment. Of these, three patients remained on therapy for more than 12 weeks (disease types: haemangioendothelioma, ovarian and adrenocortical carcinoma). The patient with advanced adrenocortical carcinoma was shown to have a differential response with reduction in size of a major metastatic lesion in the retroperitoneum, stable disease in other nodal sites but progressive disease in the liver. In light of this observation, he went on to receive a further 6 weeks of therapy; however, with further evidence of tumour progression in the liver lesions, he was withdrawn from the study. Progressive disease was the best response in 14 patients. One of these, a 53-year-old woman with advanced head and neck carcinoma, was deemed to have progressive disease having developed necrosis of the nasal area. It is possible that this represented a tumour response, but palliation was clearly not being achieved. On cessation of therapy, there was an improvement in the degree of tumour-related erythema and pain.

### Pharmacodynamics

#### VEGF

**Figure 3:**
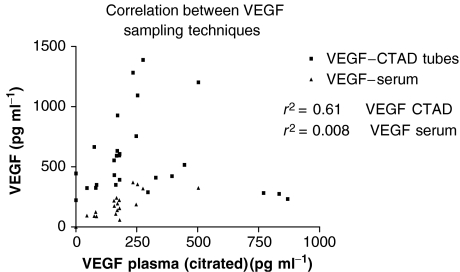
Correlation between VEGF sampling results taken from plasma, serum and platelet-stabilised plasma (CTAD).

VEGF profiles are available for nine patients in the final cohort. As expected, serum VEGF levels were universally higher than those obtained from Plasma_citrated_ or platelet-stabilised plasma (Plasma_ctad_). A significant correlation between the VEGF–Plasma_ctad_ levels and those in VEGF–Plasma_citrated_ was found (*r*^2^=0.61 *P*<0.0001); however, there was no comparable correlation with serum values (Figure 3).

**Figure 4:**
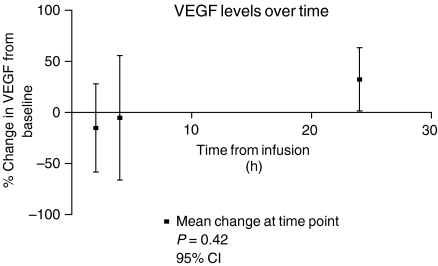
Summary of VEGF changes over time.

When the percentage change in VEGF level from baseline was assessed over time, there was a trend suggesting that the levels dropped initially with subsequent rebound. However, the sample size was small, the variance wide and the change did not reach statistical significance (Figure 4).

#### DCE-MRI

In all, 24 patients were evaluable for changes in vascular permeability. A total of 80 MRI examinations were performed in total: 24 patients at baseline; 24 patients on day 1 (3–4 h post-therapy); 17 patients were scanned mid-treatment (after 2 weeks of therapy) and 15 after more than 4 weeks (at a time point adjacent to standard tumour evaluation).

At baseline, seven patients proved unassessable. Initial tumour median transfer constant (*K*_trans_) was 0.237 min^−1^ with values ranging from 0.091 to 1.325. Median *V*_e_ was 32.5% (range 11–45%). Pretreatment kinetic parameters did not predict for eventual tumour response.

Nine patients were not evaluable on the first day of treatment. One patient, with metastatic melanoma (target lesion – brain), had a statistically significant change in *K*_trans_, 91% increase. No statistically significant changes in leakage space (*V*_e_) were seen. No dose response was seen in any parameter at this early time point.

Of the 17 patients evaluated mid-treatment, seven were unassessable. One patient with squamous cell carcinoma and disease in the nasopharynx receiving 110 mg m^−2^ showed marked reductions in *K*_trans_ (−51.9%) and *V*_e_ (−48.6%). There was no evidence of a dose response in any kinetic parameter after 2 weeks of therapy.

The median time to the final scan was 35 days (range 28–58 days). Seven of the 15 patients scanned at this time point proved assessable. Two patients had statistically significant increases in leakage space (36.5 and 34.5%). The first, a patient with soft-tissue sarcoma, treated at 48 mg m^−2^, was found to have progressive disease. The second, again soft-tissue sarcoma, was treated in the final expansion cohort, and was shown to have stable disease at the 6-week assessment. Again there was no evidence of a dose response in the kinetic parameters obtained.

**Figure 5:**
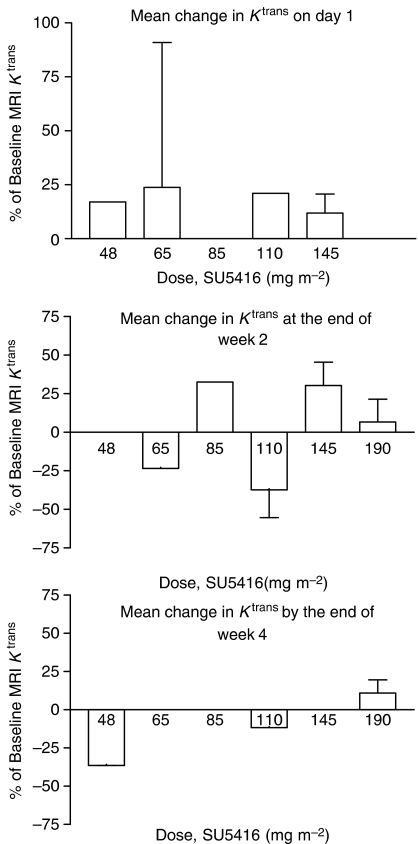
Dose-related changes in *K*_trans_ using DCE-MRI (displaying mean and standard deviation).

The changes in *K*_trans_ across all dose levels and time points are summarised graphically in Figure 5.

## DISCUSSION

A maximum-tolerated dose of SU5416 when administered as a twice weekly intravenous infusion was not identified in this study. Utilising PK observations, we have shown that it is possible to deliver 190 mg m^−2^ on a protracted basis after an introductory phase of 2 weeks therapy at a lower dose. This dose was previously determined to cause DLT. In the parallel phase, one trial conducted by Rosen *et al* (1999) using the same schedule as used here, DLTs of projectile vomiting, headache and nausea, were reported at 190 mg m^−2^. In the dose expansion cohort of our current study (145 → 190 mg m^−2^), there were patients who experienced mild headache and emesis. Clinical experience also showed that patients who had breaks of therapy longer than a week could not have treatment reinitiated at a dose of 190 mg m^−2^ without unacceptable toxicity. The 145 mg m^−2^ dose level was thus considered the recommended dose for future study.

Acute hypersensitivity reactions attributable to the diluent Cremophor® were common. However, in oncology practice such reactions are well recognised and can be easily managed. In all patients in this study, a gradual reduction in prophylactic steroid dose was possible and did not preclude sustained therapy.

The overall tolerability of intravenous SU5416, particularly when central venous access was utilised, was good. Such characteristics are important with an antiangiogenic compound such as this, where chronicity of therapy is expected and the toxicity thresholds are therefore necessarily set lower.

Homeostasis of coagulation in humans is achieved through a complex balance of plasma, tissue and endothelial factors. The process of tumoral angiogenesis mediated through the VEGF family of growth factors will have effects on vascular permeability and endothelial cell proliferation. Thus, we postulated that targeting these pathways with a drug such as SU5416 might result in physiological consequences within the coagulation pathway. Although the sample size examined was small, a consistent finding was the observation of an increase in PAI-1 levels in the acute phase (2–4 h after infusion) and a parallel reduction in PAP complexes. These changes reflect a transient effect within the fibrinolytic pathway. In those patients with additional risk factors, modulation of PAI-1 and PAP could contribute to an excessive hypercoagulable state and clinical thrombosis. This observation may relate to the concomitant administration of dexamethasone (Huang *et al*, 1995; Ha *et al*, 2002), although a relationship with SU5416 is also possible. Other than thrombophlebitis directly related to the infusion sites, only two thrombotic episodes, both venous, were observed in this study. It is important to note, however, that almost a quarter of patients received concomitant warfarin therapy, which even at low dose may have been sufficient to ameliorate this hypercoagulable propensity. An increased incidence of thromboembolic events has been reported in combination studies with SU5416 (Kuenen *et al*, 2002a; Cooney *et al*, 2005). In affected patients, Kuenen *et al* (2002b) found an increase in markers of thrombin generation and endothelial cell activation, which occurred in a cycle-dependent manner. Discerning the contribution of SU5416 alone to these findings is difficult. Although increased numbers of thrombotic events are not a feature of the parallel single-agent Phase I reports (Rosen *et al*, 1999; Stopeck *et al*, 2002), this is perhaps a feature of study size. Thrombotic events are reported in a number of the single-agent Phase II studies in the literature to date (Heymach *et al*, 2004; Peterson *et al*, 2004). Further exploration of this area is warranted and prophylactic anticoagulation is recommended in all combination studies (Cooney *et al*, 2005).

Pharmacokinetic evaluations confirmed time-dependent induction of SU5416 metabolism. In this study, the degree of induction (22%) was less than that seen in the parallel Phase I study (69%) using the same schedule (Cropp *et al*, 1999; Rosen *et al*, 1999). As the PK sampling for our current study was performed on day 4, in contrast to day 1 in the Rosen trial, it is likely that the induction in enzymes of metabolism occurs as early as the first dose. As such, the change in clearance mechanisms was probably largely complete by the time blood sampling was performed during and after the second infusion (day 4) in our study. Interestingly, the degree of induction also appears to relate to frequency of dosing as Stopeck *et al* (2002) demonstrate a loss of any induction with a treatment interval of 7 days. The mechanism for the induction in SU5416 metabolism is not understood in detail but may relate to the upregulation of cytochrome *P*450 isoenzymes (Antonian *et al*, 2000). The degree of interpatient variability in AUC is tolerable given the kinetics are linear and the toxicity profile is favourable. We believe that the variability is largely driven by interpatient differences in handling of SU5416 as the assay has been well validated and results are consistent across trials. As SU5416 in humans is metabolised through liver microsomes to at least six metabolites (Antonian *et al*, 2000), again the explanation for the degree of variability is most likely explained by differences in pharmacogenomics of cytochrome *P*450 enzymes, affecting the metabolism of SU5416 and probably influenced by drug–drug interactions. Our results show that SU5416 has a large volume of distribution and clears rapidly. This is consistent with the assessments in preclinical models (Antonian *et al*, 2000). Initial preclinical models also demonstrated that the intracellular half-life of SU5416 is long and levels capable of target inhibition are maintained for >48 h, with target inhibition resulting in successful inhibition of tumour growth (Fong *et al*, 1999), thus a twice weekly infusion schedule was recommended. *In vivo* experiments, however, do not confirm the durability of this inhibition, further emphasising the importance of developing accurate pharmacodynamic markers in the clinical development of such compounds (Mendel *et al*, 2000).

It is now generally acknowledged that serum samples are inappropriate for the measurement of VEGF, as platelets, especially when activated in clot formation, provide a rich source of this growth factor (Maloney *et al*, 1998; Wynendaele *et al*, 1999; Jelkmann, 2001). The plasma samples obtained in sodium citrate and those in the CTAD were reproducible, correlated well and one method is not preferred over the other. Although the results did not reach statistical significance, a trend was suggested with an initial drop in VEGF levels within the first 4 h following infusion, rebounding by 24 h. The timing of this observation suggests it may reflect the action of dexamethasone (Huang *et al*, 1995; Ha *et al*, 2002).

Dynamic contrast-enhanced MRI offers a unique opportunity for noninvasive functional assessment achievable on most clinically available MR systems. In a similar study with the VEGF2 and VEGF1 inhibitor PTK787/ZK22584 (Thomas *et al*, 2001; Thomas *et al*, 2002), a correlation was demonstrated between a reduction in *K*_i_ (equivalent to *K*_trans_) and both dose and plasma levels of the study compound. In this study, the tumours studied were liver metastases from colorectal cancer and patients at all dose levels administered were evaluated using DCE-MRI.

New blood vessels in tumours are structurally abnormal; leaky, with fragmented supportive structures (pericytes and basement membranes). A successful antiangiogenic therapy would therefore result in stabilisation of the vasculature, a reduction in permeability, levels of interstitial fluid hypertension and thus the size of the leakage space (Fox *et al*, 2001; Jain, 2005). Through PK modelling of the behaviour of a low-molecular weight contrast agent, DCE-MRI allows measurement of these parameters: permeability – *K*_Trans_; leakage space – *V*_e_ (Galbraith *et al*, 2002; Choyke *et al*, 2003). No dose–response changes were seen in *K*_trans_ or *V*_e_ at any of the time points assessed in our trial with SU5416. A failure to demonstrate a consistent change may reflect a number of issues. Firstly, and most likely, it may be that SU5416 is insufficiently potent enough to cause changes in vascular permeability of sufficient magnitude to be seen with this tool. Secondly, the sample size was small and as now published the 95% confidence interval for a meaningful change in *K*_trans_ in a single patient tumour is a change of −48 to +83%, and for *V*_e_ ±24% (Galbraith *et al*, 2002). It is a limitation of our study that the design did not include internal reproducibility or repeatability data for the study population. Thus, our ability to determine meaningful differences in cohorts of this size is curtailed. Thirdly, it is known that Flk-1-KDR-mediated VEGF activity is unevenly distributed within tumour vasculature (Holash *et al*, 1999; Nakopoulou *et al*, 2002). The ‘regions of interest’ chosen for evaluation in these patients may not always have been representative. Finally, the nature of the patient population in this Phase I study may have influenced the pharmacodynamic results as patients with advanced disease may have more mature vasculature that is functionally and biologically less sensitive to antiangiogenic therapy. As shown by Thomas *et al* (2001), a homogeneous population with progressive or recently emergent tumours may provide a better opportunity for demonstrating pharmacodynamic relationships than the tumours investigated in our study.

The evidence of antitumour activity is anecdotal. One patient with progressive haemangioendothelioma remained on study for 6 months with stable disease and only withdrew because personal circumstances precluded her remaining in the United Kingdom. A second patient, with squamous cell carcinoma of the nasopharynx developed necrosis of her tumour while receiving treatment. On cessation of therapy, there was clinical improvement in local induration and pain. Particularly when viewed in the context of the marked decrease in vascular permeability observed in this patient on MR, this may represent evidence of biological activity with SU5416. In early clinical studies conducted in patients with tumours characterised by particularly strong signalling through the VEGF2 receptor, for example, Kaposi's sarcoma and haemangioblastoma associated with von Hippel–Lindau syndrome, SU5416 has demonstrated encouraging activity (Miles *et al*, 2000; Aiello *et al*, 2002; Jennens *et al*, 2004). In larger Phase II studies conducted in solid tumours, the response rates have been disappointing (Heymach *et al*, 2004; Peterson *et al*, 2004; Stadler *et al*, 2004). It seems likely that although the dose level recommended by the Phase I studies achieves systemic levels that in preclinical modelling produced target and tumour growth inhibition (Fong *et al*, 1999; Mendel *et al*, 2000), it does so only transiently, and that successful therapeutic angiogenesis inhibition requires continuously above the threshold for inhibition of a critical pathway. Alternatively, the intrinsic redundancy of signalling mechanisms associated with the angiogenic process may convey tumour resistance to antiangiogenic compounds acting mainly on a single target (Kanno *et al*, 2000; Fox *et al*, 2001). Further trials using combinations of therapies in this class or kinase inhibitors with broader target specificities are required.

A twice weekly infusion of SU5416 is safe and able to be delivered in a protracted manner. Treatment can achieve potentially therapeutic systemic levels of SU5416. Understanding the PK behaviour of the drug has allowed us to develop an intrapatient dose escalation schedule (145 mg m^−2^ twice weekly for 2 weeks, increasing to 190 mg m^−2^ if well tolerated), which overcomes the lowering of plasma concentration resultant from induction of drug metabolism. This trial failed to demonstrate a pharmacodynamic effect using DCE-MRI, but does illustrate some of the challenges inherent in conducting such studies. Incorporation of DCE-MRI in further studies with antiangiogenic therapy is important for the validation of this tool, but we would suggest that such studies include an intrapatient baseline assessment of reproducibility and cohort sizes of sufficient number to determine meaningful differences.

## Figures and Tables

**Figure 1:**
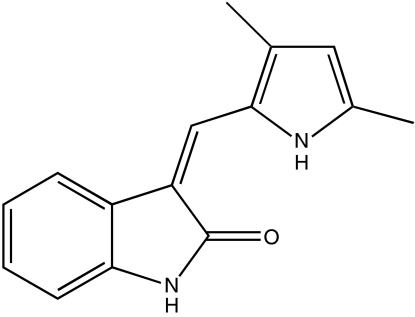
SU5416 chemical structure, 3-[(2,4-dimethylpyrrol-5-yl)methylideneindolin-2-one.

**Table 1:** Patient characteristics

	**Patient characteristics**
Age	Median 48 years
	Range 18–74 years
Gender	14 males and 14 females
	
*Tumour types*	
Soft-tissue sarcoma	10
Ovary/cervix/endometrium	4
Melanoma	3
Renal	2
Head and neck	2
Other (one each)	Osteosarcoma, adrenocortical, small cell, transitional cell, gastro/oesophageal junction, Ca unknown primary

**Table 2:** Pharmacokinetic results

**Patient**	**Dose (mg m^−2^)**	**Cycle**	***C*_max_ (*μ*g l^−1^)**	**AUC_last_ (h^a^** ***μ*g l^−1^)**	***T*_1/2_*λ****_z_* **(h)**	**CL (observed) (l h^−1^)**	***V_z_* (observed) (l)**
1	48	1inj2	1737	463	0.54	181	142
2	48	1inj2	2724	848	0.51	94	69
		1inj8	2059	630	0.61	125	109
		2inj8	1629	498	0.57	158	131
3	48	1inj2	2253	1143	0.61	83	72
		1inj8	1289	816	0.42	115	69
4	65	1inj3	2074	566	0.20	190	55
5	65	1inj2	4371	1498	0.45	64	41
		1inj8	4105	1312	0.73	74	78
7	65	1inj2	2968	2510	0.82	74	88
		1inj8	2914	2068	0.65	89	84
8	85	1inj2	2335	1809	0.39	79	45
		1inj8	3672	2375	0.60	60	52
		2inj8	2804	1619	0.74	88	94
9	85	1inj2	1624	1151	0.71	133	136
10	85	1inj2	2057	2094	0.71	102	104
		1inj8	1354	1250	2.05	151	447
11	110	1inj2	3507	4016	0.62	46	41
12	110	1inj2	2750	2345	0.69	70	70
13	110	1inj2	3237	3842	0.55	83	66
		1inj8	3578	3796	0.62	84	75
14	145	1inj2	4851	4946	0.87	55	69
		1inj8	3922	4428	0.57	62	51
15	145	1inj2	2869	3471	0.89	92	119
16	145	1inj2	1217	2021	0.71	150	153
		1inj8	2080	2349	0.67	169	164
		2inj8	1342	1779	0.72	224	233
17	145 → 190	1inj2	4159	3389	0.68	64	63
		1inj8	3370	4467	0.62	63	57
18	145 → 190	1inj2	2474	1928	0.50	96	70
		1inj8	2274	2390	0.50	103	74
		3inj2	6517	7173	0.75	44	48
19	145 → 190	1inj2	2961	3683	0.79	66	75
		2inj1	4002	6451	0.77	49	54
20	145 → 190	1inj2	2774	3397	0.66	89	85
		1inj8	2066	2611	0.81	151	176
		2inj8	3341	5044	0.72	79	82
21	145 → 190	1inj2	2894	2621	1.36	100	196
		1inj8	2161	2178	2.38	142	488
22	145 → 190	1inj2	2795	2870	0.67	101	97
23	145 → 190	1inj2	3202	2465	1.15	72	119
24	145 → 190	1inj2	1177	1267	0.38	215	118
		1inj8	493	816	0.42	352	214
25	145 → 190	1inj2	4508	4094	0.49	55	39
		1inj8	4283	5360	0.58	56	46
27	145 → 190	1inj2	2625	1578	0.69	106	106

*C*_max_=maximum concentration; *T*_max_=time of maximum observed concentration, AUC_last_=area under the curve from time of dosing to the last measurable concentration; AUC_inf_=area under the curve from the time of dosing to infinity; *t*_1/2_*λ_z_*=terminal half-life ln_2_/*λ*_z_, where *λ*_z_ is the first-order rate constant associated with the terminal log-linear portion of the curve; MRT_last_=mean residence time from the time of dosing to the last measurable concentration; *V_z_*=volume of distribution based on the terminal phase dose/λ_z_^*^AUC_inf_; CL=total body clearance.

a Patient 6 and 26 – not evaluable for PK, samples not taken.
